# Mechanical prophylaxis for venous thromboembolism prevention in obese individuals

**DOI:** 10.1177/02683555211031147

**Published:** 2021-07-06

**Authors:** Amulya Khatri, Alun H Davies, Joseph Shalhoub

**Affiliations:** 1 Academic Section of Vascular Surgery, Department of Surgery & Cancer, Imperial College London, London, UK; 2 Imperial Vascular Unit, Imperial College Healthcare NHS Trust, London, UK

Obesity represents a significant risk factor for venous thromboembolism (VTE), with a relative risk increase of 2.5 for the development of deep vein thrombosis (DVT).^1^ Given the rise in rates of obesity,^2^ targeted efforts are necessary to refine and optimise VTE prophylaxis regimens for obese individuals.

Mechanical and pharmacological prophylaxis remain the mainstay in VTE prevention. Current mechanical strategies to reduce venous stasis include graduated compression stockings (GCS) and active devices, such as intermittent pneumatic compression (IPC).^3^ Whilst mechanical prophylaxis is effective in preventing VTE compared to no intervention, its clinical utility has been cast into doubt, particularly as an adjunct to other strategies. Recently the PREVENT and GAPS trials, published in 2019 and 2020, respectively, showed no added benefit of mechanical prophylaxis when pharmacological prophylaxis was in place.^4,5^

The addition of pharmacological to mechanical prophylaxis with IPC, and vice versa, was the subject of a 2016 Cochrane Review.^6^ The meta-analysis performed showed that the addition of IPC to pharmacological prophylaxis reduced episodes of PE, with no difference in DVT or bleeding. However, the addition of pharmacological prophylaxis to IPC reduced the incidence of DVT at a cost of increased risk of bleeding, without impacting upon rates of pulmonary embolism (PE).^6^ A subsequent trial by Kamachi et al., which compared IPC with IPC plus pharmacological prophylaxis, found the addition of pharmacological prophylaxis yielded no difference in rates of either DVT or PE;^7^ it being noteworthy that all VTE episodes in this study were asymptomatic and detected on screening CT imaging. These findings in this recent trial may be driven, at least in part, by the wide-reaching advances in patient care that have been observed to drive down VTE rates, such as early mobilisation and enhanced recovery after surgery protocols, which may have contributed to underpowering in this study.^8^

Importantly, obese patients have been relatively underrepresented in past trials^9^ with, for example, obese patients’ inclusion ranging between 2.9% and 42% in the above trials.^7,10^ Impaired venous return worsened by increased intra-abdominal pressure, longer hospital admission and reduced mobility are proposed mechanisms for the increased risk of VTE in the obese patient group.^11^ Co-morbidities associated with obesity, such as varicose veins or impaired cardiac function, may further worsen venous return. As such, it is plausible that obese patients may particularly benefit from mechanical prophylaxis to counteract these contributors to venous stasis. Indeed, counteracting the immobility in stroke patients with IPC significantly reduced VTE incidence in the CLOTS 3 trial.^12^

Despite obesity being a VTE risk factor and the theoretical benefit of mechanical prophylaxis, there remains a lack of evidence in this area. This paucity of data was highlighted almost a decade ago by Freeman and colleagues yet, to the best of our knowledge, no trials have been initiated focusing on obese patients.^13^

A systematic database search performed by the authors of this editorial, combining relevant free text and MeSH terms relating to VTE, mechanical prophylaxis and obesity, found no studies investigating mechanical prophylaxis in obese patients outside of bariatric surgery, despite the number of trials investigating VTE prophylaxis in general. The use of mechanical prophylaxis in bariatric surgery was the subject of a recent systematic review.^14^

Limited evidence may be extrapolated from two observational studies investigating bariatric surgical patients, comparing IPC against the combination of IPC and pharmacological prophylaxis. Whilst Gagner et al. found no significant difference between these two regimens, suggesting IPC may be sufficient for VTE prophylaxis, the study was limited by a low number of participants in the IPC only arm, lack of randomisation and relatively low rates of VTE compared to those reported in the literature.^15^ Frantizides et al. found that IPC combined with early mobilisation and emphasis on hydration reduced the incidence of VTE compared to IPC with routine pharmacological prophylaxis, with an increased rate of bleeding in the latter group.^16^ Amongst this study’s limitations were higher BMIs and longer operating times in the group receiving the combination of IPC and pharmacological prophylaxis.^16^

The use of IPC with early ambulation was able to achieve low rates of DVT occurrence (3/957, 0.31%) without the use of pharmacological prophylaxis, suggesting IPC to be an effective method in obese patients. Despite the promising results, the single regimen impairs our ability to assess the impact of mechanical prophylaxis in the context of other interventions, such as early ambulation on the first post-operative day.^17^

Current European Society of Anaesthesiology guidelines on VTE prophylaxis in obese patients relies on the above discussed literature, resulting in limited recommendations due to the lack of high-quality evidence.^18^

Outside of bariatric surgery, there continues to be a dearth of data. In 2007, Turpie and colleagues conducted a randomised controlled trial showing the addition of pharmacological prophylaxis to IPC reduced VTE events, but found no significant difference within obese patients in sub-group analysis.^10^ However, the large confidence intervals point to the limited power of this sub-group analysis and, in turn, restrict the conclusions which can be drawn.

Therefore, further research is required to investigate whether differential effects of mechanical prophylaxis in obese patients exist, extending beyond patients undergoing surgery for weight loss. Sub-group analysis from existing trials may provide initial data, providing preliminary evidence to inform and facilitate the design of further studies. Future studies, if not specifically focused on obese patients, should consider including obese patients as an appropriately powered *a priori* subgroup.

The assumption that all mechanical prophylaxis produces a homogenous effect may impair any conclusions drawn from these studies. Whereas GCS provides continuous compression, IPC employs active intermittent compression more comparable to ‘muscle pump’ physiology. This appears to translate into clinical utility, with the CLOTS 3 trial finding a benefit of IPC in newly admitted stroke patients, whilst the CLOTS 1 trial found GCS to have no benefit in VTE prophylaxis.^12,19^ Previous meta-analyses of hospitalized and of critically ill patients have also suggested IPC to have greater efficacy than GCS.^3,20^

The location of IPC device placement also appears to impact the prevention of DVT, and haemodynamic studies have shown variation in venous blood flow depending on the method and brand of IPC system in place.^21–23^ Thus, comparing the efficacy of various aspects of GCS and IPC use in obese patients will also be required to truly assess the role of mechanical prophylaxis in this population. Consideration must also be given to the difficulties in applying mechanical prophylaxis to obese legs and the associated financial and personnel resources, impacting adherence with and effectiveness of compression in clinical practice.

Finally, the optimal regimen for pharmacological prophylaxis in obese patients is yet to be determined and has been reviewed elsewhere.^24^ The authors again note that obese patients are underrepresented within these clinical trials, resulting in an unclear optimal regimen for various heparins and direct oral anticoagulants.

There remains a lack of evidence to determine the optimal VTE prophylaxis regimen in obese individuals, calling for appropriately designed and powered clinical studies specifically aimed at this important group.
